# Concurrent neoadjuvant endocrine therapy with chemotherapy in HR+HER2- breast cancer: a systematic review and meta-analysis

**DOI:** 10.3389/fendo.2024.1254213

**Published:** 2024-02-28

**Authors:** Ping Wu, Wenjie Lv

**Affiliations:** Department of Breast Surgery, Xinhua Hospital Affiliated to Shanghai Jiao Tong University School of Medicine, Shanghai, China

**Keywords:** concurrent, neoadjuvant endocrine therapy, chemotherapy, HR+HER2-breast cancer, meta-analysis

## Abstract

The role of simultaneous neoadjuvant endocrine therapy in chemotherapy in HR+HER2- breast cancer continues to be controversial. This systematic review and meta-analysis was conducted to further evaluate the effectiveness and safety of this strategy for HR+HER2- breast cancer patients. Trials in which HR+HER2- breast cancer patients were randomly assigned to either single or simultaneous endocrine-assisted neoadjuvant chemotherapy were eligible for inclusion. The prime endpoint was the pathological complete response (pCR) rate. The clinical response (complete clinical response: CR, partial response: PR) and safety were secondary endpoints. A random effect model was used for statistical analysis. A total of 690 patients from five trials were included. PCR rate was 10.43% in the concomitant endocrine group and 7.83% in control group (OR=1.37, 95%CI 0.72-2.60, P=0.34). The CR rate was 15.50% for the concomitant endocrine group and 10.26% for the control group. (OR=1.61, 95%CI 0.99-2.61, P=0.05). ORR (CR+PR) was significantly higher in the simultaneous endocrine group compared to the control group (79.53% (272/342) vs. 70.09% (239/341) , OR=1.70, 95%CI 1.19-2.43, P=0.004) and the meta-analysis approach showed no heterogeneity (I^2 =^ 0%, P=0.54) . Tamoxifen concurrent with chemotherapy could increase the frequency of adverse events, whereas aromatase inhibitors (AIs) would not. Our findings provide evidence for the efficacy and safety of concurrent neoadjuvant endocrine therapy (AIs) with chemotherapy as an available option to achieve a higher clinical response rate for HR+HER2- breast cancer patients compared with chemotherapy alone with low toxicity.

**Systematic review registration:**

https://www.crd.york.ac.uk/PROSPERO/, identifier CRD42022340725.

## 1 Introduction

Neoadjuvant therapy has become the standard strategy for patients with locally advanced breast cancer. Pathologic complete response (pCR) to preoperative systemic therapy is associated with an extremely favorable disease-free and overall survival (1, 2).International guidelines recommend that a neoadjuvant approach be preferred in subtypes highly sensitive to chemotherapy, such as triple-negative and HER2+ (3–6). HR+HER2- carcinomas are generally less responsive to primary chemotherapy and may benefit less in neoadjuvant setting. Neoadjuvant endocrine therapy for this subtype is also not recommended by guidelines due to limited therapeutic efficacy. Small sample clinical trials have suggested equal rates of clinical response for endocrine therapy as for chemotherapy, though neither approach routinely achieves a rate of pCR>10% (7, 8).

Therefore, neoadjuvant concurrent endocrine therapy with chemotherapy for this particular type of tumor is worth further investigating, while whether concurrent endocrine therapy with chemotherapy in neoadjuvant setting can be of real clinical benefit has never been elaborated. Endocrine therapy needs to be sequenced after chemotherapy based on the previous understanding of adjuvant therapy. However, the prime purpose of neoadjuvant therapy is to shrink the tumor as early as possible with powerful regimens, and delaying endocrine therapy may deprive the patient of the best opportunity for treatment. The previous study suggests in patients with potentially hormone-sensitive metastatic breast cancer, chemohormonal therapy prolongs the time to treatment failure (TTF) for ER-positive patients (9). There are few studies of concurrent endocrine therapy with chemotherapy in neoadjuvant setting worldwide, as well as a lack of systematic reviews to objectively assess the efficacy of their concurrent treatment. This study seeks to provide more conclusive clinical evidence on this controversial topic by conducting a systematic review and meta-analysis on the data from randomized trials that investigated the role of concurrent endocrine therapy as a feasible strategy in neoadjuvant setting for patients with HR+Her2- breast cancer.

## 2 Methods

This systematic review and meta-analysis was reported in accordance with the Preferred Reporting Items for Systematic Reviews and Meta-analysis guidelines (10, 11). A protocol was developed prior to the survey launch and presented to PROSPERO. (registration number CRD42022340725).

### 2.1 Search strategy

The PubMed, Embase, and Cochrane Library electronic medical publication databases were searched for relevant randomized controlled trials (RCTs) released before June 2022. Potential relevant RCTs were identified through various combinations of the following search terms: breast neoplasms, concurrent, neoadjuvant therapy and endocrine therapy.

### 2.2 Selection criteria

Randomized controlled trials that enrolled patients of HR+ HER2- breast cancer in neoadjuvant setting were included. In addition, studies were considered relevant if (a) the study concerned clinical research comparing concurrent chemo-endocrine therapy versus chemotherapy alone, (b) the pCR rates and clinical responses had to be reported, (c) the manuscript was published in English, (d) with full text available. Trials that only studied ovarian suppression were excluded. In-progress trials that have not yet been presented at conferences or published or available online at the time of the literature search have also been excluded.

Patients of HR+ HER2- breast cancer enrolled in the study were required to receive chemotherapy and endocrine therapy in neoadjuvant setting. Chemotherapy regimens include EC/AC-T (epirubicin/doxorubicin+ cyclophosphamide four cycles followed by docetaxel four cycles), TAC (epirubicin/doxorubicin+ cyclophosphamide+ docetaxel four cycles), TP (albumin paclitaxel+ carboplatin/cisplatin),CMF (cyclophosphamide +methotrexate+5-fluorouracil) and FEC (5-fluorouracil+epirubicin+cyclophosphamide). Endocrine therapy includes tamoxifen/aromatase inhibitor ± ovarian suppression.

### 2.3 Data extraction

This study aimed to evaluate both the efficacy and the safety of neoadjuvant chemotherapy with or without concurrent endocrine of patients with HR+ HER2- breast cancer. Primary end point was pCR rate. Secondary end points were clinical response and safety. For each eligible study we collected study design, number of patients enrolled overall and into the two study arms. Menopausal status, type of chemotherapy and endocrine therapy administered, the number of patients who achieved pCR and CR (complete response) or PR (partial response), toxicity and adverse events were also collected in both study arms.

Data from each of the included tests were thoroughly verified to ensure that they were consistent with their original publications. The discrepancies were discussed and resolved with the authors prior to aggregation into the final unified database used for the analysis.

### 2.4 Statistical analysis

All analyses were completed, including the total number of patients for which information was available for each specific endpoint. Odds ratios (ORs) with 95% confidence intervals [CI] comparing concurrent administration of neoadjuvant endocrine therapy with chemotherapy and chemotherapy alone were calculated from each article and the synthetic risk estimation was calculated using the DerSimonian and Laird Random Effect Model (12). OR < 1 indicated better outcome for chemotherapy arm, OR > 1 indicated favored prognosis for concurrent arm. Heterogeneity among studies was quantified by the Higgins I^2^ index.

All statistical tests were two-sided, with P <.05 values considered statistically significant. Statistical analyses and the generation of forest plot were carried out by Reviewer Manager 5.3.

## 3 Results

**Figure 1:**
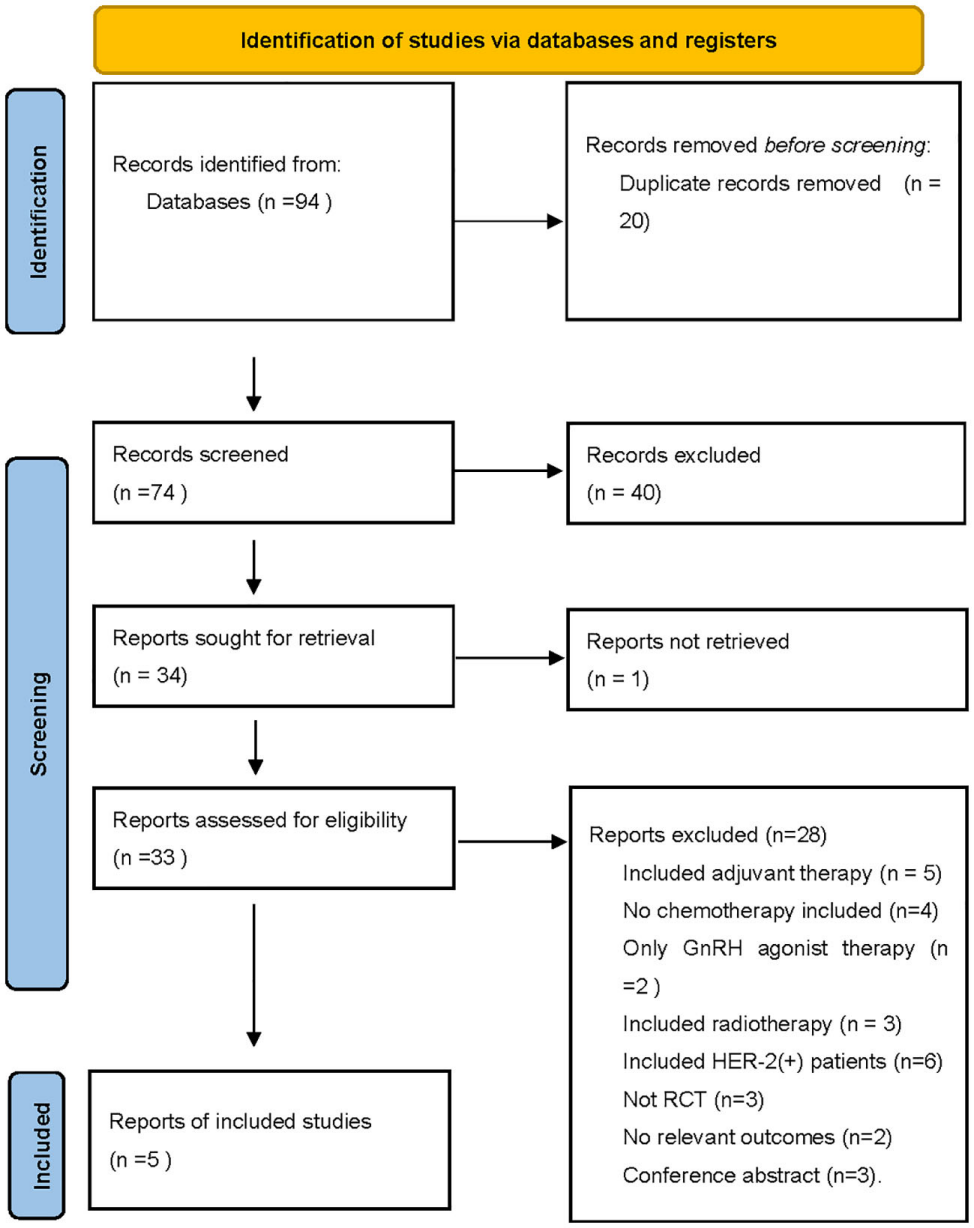
The PRISMA flow chart summarizing the process for the identification of the eligible studies.

Of the 94 entries returned during the initial database search, 89 were excluded because they failed to meet the inclusion criteria. In total, five separate randomized trials were considered eligible for this study (13–17). (**Figure 1**) Mohammadianpanah et al. (2011) and K. Sugiu et al. (2015) trials included 24 and 5 HER2+ patients, respectively. With no neoadjuvant targeted therapy, the findings were valid and the studies were included. Two studies (M. Mohammadianpanah (2011) and G. Minckwit (2001)) enrolled some ER- patients, balanced in both studies, hence also included.

In total, 690 patients were included, of which 345 were randomized to the study group (concurrent endocrine with chemotherapy) and 345 to the control group. Two of the five studies included ER(-) patients. Tamoxifen was used for endocrine therapy in 1 study, and aromatase inhibitors (AIs) were used in the rest. All premenopausal patients were given ovarian function suppression (goserelin/leucovorin) when AIs were administered. **Table 1** summarizes the main characteristics of the five included trials.

**Table 1:** Characteristics of the five included randomized trials.

Study	R.Matsunuma, 2020 (13)	K-D Yu, 2019 (14)	G.Minckwitz,2001 (15)	K.Sugiu, 2015 (16)	M. Mohammadianpanah, 2011 (17)
Country	Japan	China	German	Japan	Iran
Sample size	70	249	247	28	96
Control/Study	35/35	124/125	125/122	12/16	49/47
ER(-)	0	0	80	0	33
Menopausal status(pre/post)	39/31	169/80	134/113	12/16	0/96
Control arm	P-AC or EC	EC-T or FEC	AT	T-FEC	FAC
Study arm	+anastrozole ± leuprorelin	+letrozole ± leuproelin	+tamoxifen	+AI ± goserelin	+letrozole
Primary endpoint	pCR rate	ORR (CR+PR)	pCR rate	pCR rate	pCR rate
Secondary endpoints	Clinical response rate, toxicity, and health-related quality of life	Ki67,pCR rate,PFS,safety	Tumor regression	Tumor regression	CR rate

P-AC, paclitaxel followed by doxorubicin; EC, epirubicin plus cyclophosphamide; FEC, fluorouracil, epirubicin, cyclophosphamide; AT, doxorubicin plus docetaxel; T-FEC, paclitaxel followed by fluorouracil, epirubicin, cyclophosphamide; FAC, fluorouracil, doxorubicin, cyclophosphamide.

### 3.1 PCR rates

**Figure 2:**
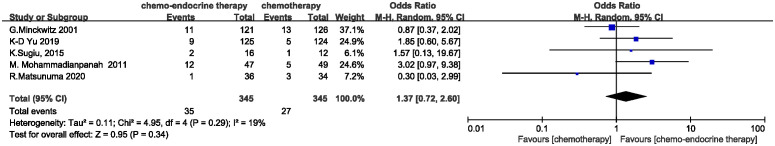
Odds ratio for pCR rate in the control arm versus concurrent administration of chemotherapy and endocrine therapy. The squares on the odds ratio plot are proportional to the weight of each study. CI, confidence interval.

All 690 patients were evaluated with pCR rates. The concurrent group has a slightly higher pCR rate than the control group (10.43%, 36/345 vs. 7.83%, 27/345), but the ORs did not reach significance (OR=1.37, 95%CI 0.72-2.60, P=0.34). (**Figure 2**) Of note, even in a study (M. Mohammadianpanah, 2011) including ER(-) patients, concurrent neoadjuvant endocrine therapy (AIs) still could achieve a higher pCR rate compared with chemotherapy alone (25.5% vs.10.2%, P=0.049). This indicated that even PR(+) patients might still benefit from endocrine therapy in neoadjuvant setting which was consistent with the current findings in adjuvant setting (18), although M. Mohammadianpanah believed ER status could be a potential predictor of a better clinical response.

### 3.2 Clinical response rates

**Figure 3:**
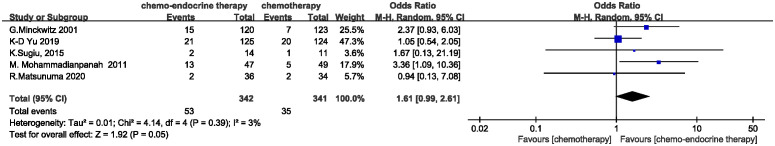
Odds ratio for CR rate in the control arm versus concurrent administration of chemotherapy and endocrine therapy.

**Figure 4:**
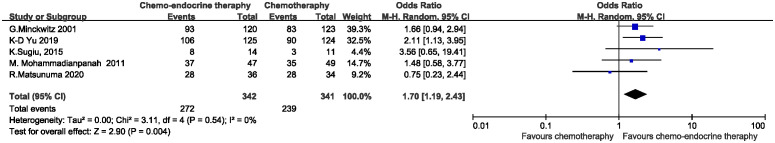
Odds ratio for ORR in the control arm versus concurrent administration of chemotherapy and endocrine therapy.

Clinical response rates were evaluated in 683 of 690 patients. The complete response (CR) rate is 15.50% (53/342) in concurrent group and 10.26% (35/341) in control group. A trend, albeit non-significant, appears to favor the concurrent addition of endocrine therapy to chemotherapy for a higher CR rate (OR=1.61, 95%CI 0.99-2.61, P=0.05) and the heterogeneity was low (I^2 =^ 3%, P=0.39) . (**Figure 3**) When the objective clinical response rates (ORR=CR+PR) were compared between the two groups, concurrent endocrine therapy could achieve a significantly higher ORR than chemotherapy alone (79.53% (272/342) vs. 70.09% (239/341) , OR=1.70, 95%CI 1.19-2.43, P=0.004) and the meta-analysis approach showed no heterogeneity (I^2 =^ 0%, P=0.54. (**Figure 4**) Two studies assessed the relationship between baseline Ki67 levels and clinical response. K-D Yu (14) found patients with a high baseline Ki-67 (>20%) demonstrated a significantly better clinical response to the concurrent treatment (91.2% vs. 68.7%, P = .001). K.Sugiu (16) showed in the high-Ki67 group, both the concurrent-therapy (P=0.084) and chemotherapy-only groups (P=0.026) had relatively favorable decreases in tumor size.

Could higher clinical response rates result in higher breast conservation (BC) rates? Three studies evaluated BC rates of the patients. G.Minckwitz (15) assessed the overall BC rates and found the rate was identical in the two treatment groups (68.6% and 69.0%, with a 95% CI for the difference of -12.0% to+11.1%). The likelihood of retaining the breast in larger tumors was highly dependent on the clinical reaction to preoperative chemotherapy. Patients with tumors greater than 4 cm had a higher rate of breast conservations if they obtained a favorable remission. R.Matsunuma (13) and K.Sugiu (16) analyzed the BC rates of the patients who were considered ineligible for breast-conserving surgery at baseline. In the former study, 22 patients were able to undergo achieved BC surgery through preoperative therapy, 13 (59.1%) of whom received concurrent therapy and 9(40.9%) of whom received chemotherapy alone. In the latter study, of the 6 patients with BC surgery, 4 (66.7%) received concurrent therapy, and 2 (33.3%) received chemotherapy alone. Both studies fully demonstrated the benefit of concurrent endocrine therapy with chemotherapy over chemotherapy alone in further improving BC rates in patients ineligible for breast-conserving surgery prior to treatment.

### 3.3 Safety

A total of 625 patients in four studies (13–15, 17) were evaluated for safety. Hematologic toxicity such as leukopenia or neutropenia was seen in all studies. The decrease in leukocytes and neutrophils was more pronounced in concurrent tamoxifen group (15), while concurrent AIs would not increase hematologic toxicity. The most common grade ≥3 adverse events for non-hematologic toxicity were gastrointestinal effects, pruritus and peripheral neuropathy, rates of which did not significantly differ between the 2 treatment groups. The majority of endocrine-related adverse events, including hot flashes and musculoskeletal pain, were mild to moderate. One study (n=96) found the concurrent arm was associated with higher rate (23.4% vs. 6.1%, P = 0.016) of hot flushes compared with the control arm (17). In one study (15) (n=237) of concurrent tamoxifen, left ventricular ejection fraction fell after treatment. Three of these four events were associated with tamoxifen. Thromboembolic events were reported in 5 patients, of which 4 were on tamoxifen therapy. On the whole, concurrent endocrine therapy would not dramatically increase rates of serious adverse reactions (Grade 3 or 4) compared to chemotherapy alone.

## 4 Discussion

This meta-analysis of five trials investigated the role of additional concurrent endocrine therapy during chemotherapy for patients with HR+ HER2- breast cancer in neoadjuvant setting. Concurrent administration of endocrine and chemotherapy could significantly increase the clinical response rate with low toxicity.

The patients with HR+ HER2- breast cancer are less sensitive to neoadjuvant chemotherapy with a low pCR rate and lack of effective treatment. The efficacy of concurrent addition endocrine therapy to chemotherapy remains controversial. The timing of endocrine administration in neoadjuvant setting currently continues to refer to the adjuvant treatment. International guidelines still recommend sequential endocrine therapy (tamoxifen or aromatase inhibitors) after chemotherapy (3, 4). The latest edition of NCCN and ESMO guidelines both recommend sequential endocrine therapy based on the same phase 3, 3-arm RCT study (19) (tamoxifen alone, sequential tamoxifen with chemotherapy, concurrent tamoxifen with chemotherapy), in which 1477 patients were eligible for analysis after a maximum of 13 years of follow-up (median 8.94 years). The study confirmed therapy with chemotherapy plus tamoxifen combined (sequential or concurrent) was superior to tamoxifen alone for disease-free survival (DFS) (adjusted Cox regression hazard ratio [HR] 0.76, 95% CI 0.64–0.91, p=0.002) and marginally for overall survival (OS) (HR 0.83, 0.68–1.01, p=0.057). The adjusted HRs preferred sequential over concurrent but did not achieve significance for DFS (HR 0.84, 0.70–1.01, p=0.061) or OS (HR 0.90, 0.73–1.10, p=0.30). Similarly, the GEICAM 9401 study (20) compared the clinical benefit of sequential versus concurrent tamoxifen with chemotherapy. No significant difference in DFS at 5 years was found between the two groups with 70% in concurrent and 75% in the sequential group (adjusted HR 1.11, 95% CI 0.71–1.73, P = 0.64). Both studies failed to show an advantage of sequential over concurrent tamoxifen with chemotherapy. Aromatase inhibitor (AI) has emerged as an option for endocrine therapy in recent years, and AI is significantly superior to tamoxifen in both neoadjuvant and adjuvant settings (21–26), suggesting that there may be a potential benefit of concurrent AI administration in the neoadjuvant phase.

The pCR rate has been recognized as an indicator of efficacy after neoadjuvant therapy. The US Food and Drug Administration and European Medicines Agency support the use of pCR in early-stage neoadjuvant breast cancer randomized trials as a surrogate for long-term patient clinical outcomes, in the accelerated approval process of new drugs (26, 27). Almost all of the studies included in this meta-analysis investigated pCR rates as the primary endpoint, and the results indicated that concurrent therapy did not significantly improve pCR rates compared with chemotherapy alone. However, the value of pCR rates in predicting prognosis is controversial. The pCR rate to preoperative systemic therapy was previously believed to be associated with an extremely favorable disease-free and overall, but the correlation between pathological response and long-term outcome was highest for triple negative breast cancer (TNBC), slightly less for HER2-positive disease, and least for ER-positive disease (1, 2). Recent meta-analysis (28) confirmed that the weak relationship between pCR and long-term clinical outcomes was evident across all subgroups studied, and pCR should not be recommended as a surrogate for prognosis. Therefore, pCR may not be an appropriate primary endpoint to assess efficacy in neoadjuvant trials of HR+ HER2- breast cancer.

In this study, we assessed the clinical response as the secondary endpoint, and found that the concurrent therapy was more likely to achieve CR than chemotherapy alone, although a statistical difference was not yet reached; however, the concurrent therapy could achieve significantly high ORR than chemotherapy alone. This suggests that concurrent therapy can lead to higher clinical response rates than chemotherapy alone, furthermore, may result in improving BC rates for patients ineligible for breast-conserving surgery at baseline. Unfortunately, only two of the five studies with small samples explored the BC rates for patients ineligible for breast-conserving surgery at baseline, and further studies with large samples may still be needed. A high Ki67 level may serve as a predictor of good clinical response. Meanwhile, one study (14) showed that patients with a higher Ki67 level at baseline were more likely to benefit from disease-free progression survival (PFS rate) with concurrent therapy than with chemotherapy alone (2-year PFS rate 91.5% vs 76.5%, P=0.058), whereas patients with a lower Ki67 level did not (P=0.317). However, whether higher clinical response rates can lead to longer survival benefits was not mentioned in all studies and further investigation is needed.

The previous belief that tamoxifen could increase the incidence of thrombotic events and cardiovascular disease (18, 29) was similarly confirmed in the only study in which tamoxifen was used (15). Concurrent tamoxifen appears to be more prone to occur these adverse events than chemotherapy alone (3/4 vs. 1/4; 4/5 vs. 1/5), and hematologic toxicity was more pronounced in the concurrent tamoxifen group. While concurrent AI as endocrine therapy did not show serious endocrine-related adverse effects (≥ grade 3), and its symptoms such as hot flashes and bone pain were mild or moderate and well-tolerated for patients. Therefore, concurrent tamoxifen seems to increase the incidence of adverse events, while concurrent AI therapy is safe and low-toxicity.

Some limitations of the current study should be acknowledged. Sample sizes were uneven, with some studies having small sample sizes which appear to be inadequate by today’s standard to make definitive conclusions. K.Sugiu et al. (2015) trial had a low sample size (n=28) and an imbalanced group allocation (only 1 T3 and 2 T4 tumors were included in the chem-endo group and none in the chemo-group). Some individual study was dated and did not use ovarian function suppression in premenopausal patients, which may have affected the efficacy of endocrine therapy. Also, since this study was not based on individual patient-level data, it was impossible to obtain treatment data for patients with different menopausal statuses, to perform subgroup analysis, and to analyze whether menopausal status affected the efficacy of concurrent endocrine therapy.

In conclusion, our systematic review and meta-analysis provides evidence for the efficacy and safety of concurrent endocrine therapy for patients of HR+HER2- breast cancer in neoadjuvant setting. The pCR rate should not be the primary end point for this subtype of breast cancer. Given the findings of our study, concurrent endocrine therapy should be considered as an available option to improve clinical response, or even maybe increase breast conservation rate. Concurrent AI therapy is safe and well-tolerated, without a significant increase in adverse effects. Further studies are still needed to investigate whether long-term survival benefits can be achieved from concurrent endocrine therapy in neoadjuvant setting.

## Data availability statement

The original contributions presented in the study are included in the article/supplementary material. Further inquiries can be directed to the corresponding author.

## Author contributions

PW: Data curation, Writing – original draft. WL: Conceptualization, Formal analysis, Investigation, Supervision, Writing – review & editing.
